# Supplementary figures and images for: Negative air ions through the action of antioxidation, anti-inflammation, anti-apoptosis and angiogenesis ameliorate lipopolysaccharide induced acute lung injury and promote diabetic wound healing in rat
Source: PLoS One. 2022 Oct 26;17(10):e0275748. doi: 10.1371/journal.pone.0275748 (PMC9604953; doi:10.1371/journal.pone.0275748)

### Figure 3A

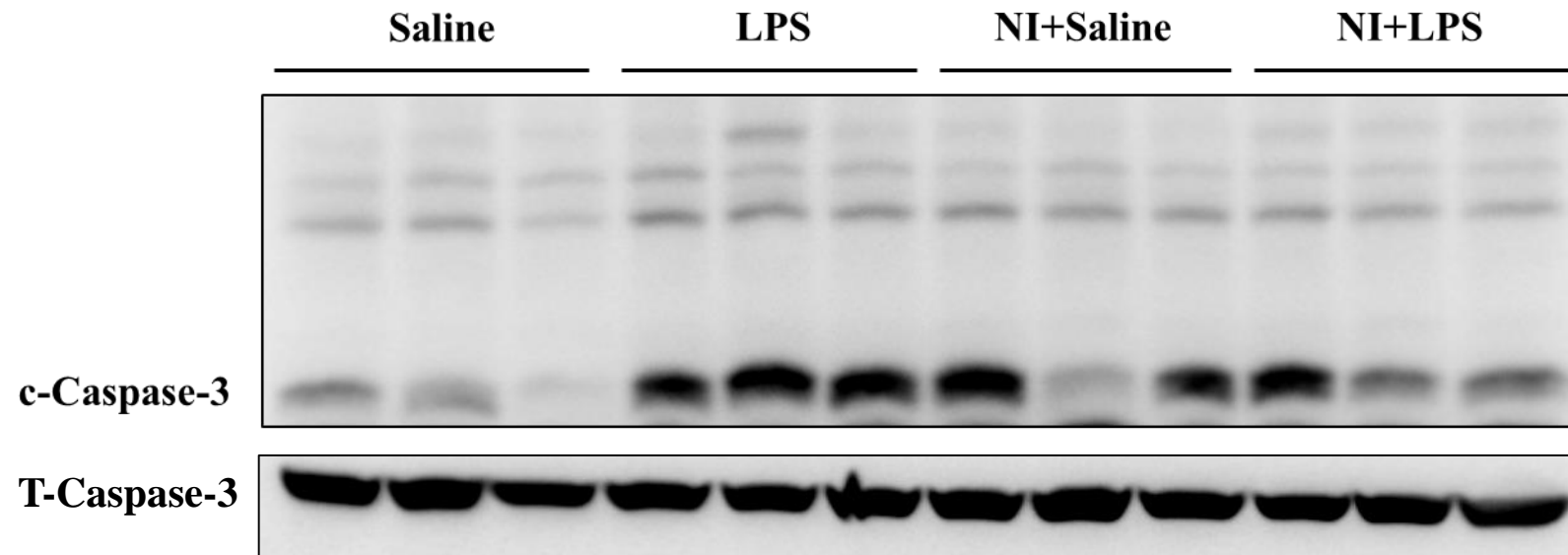

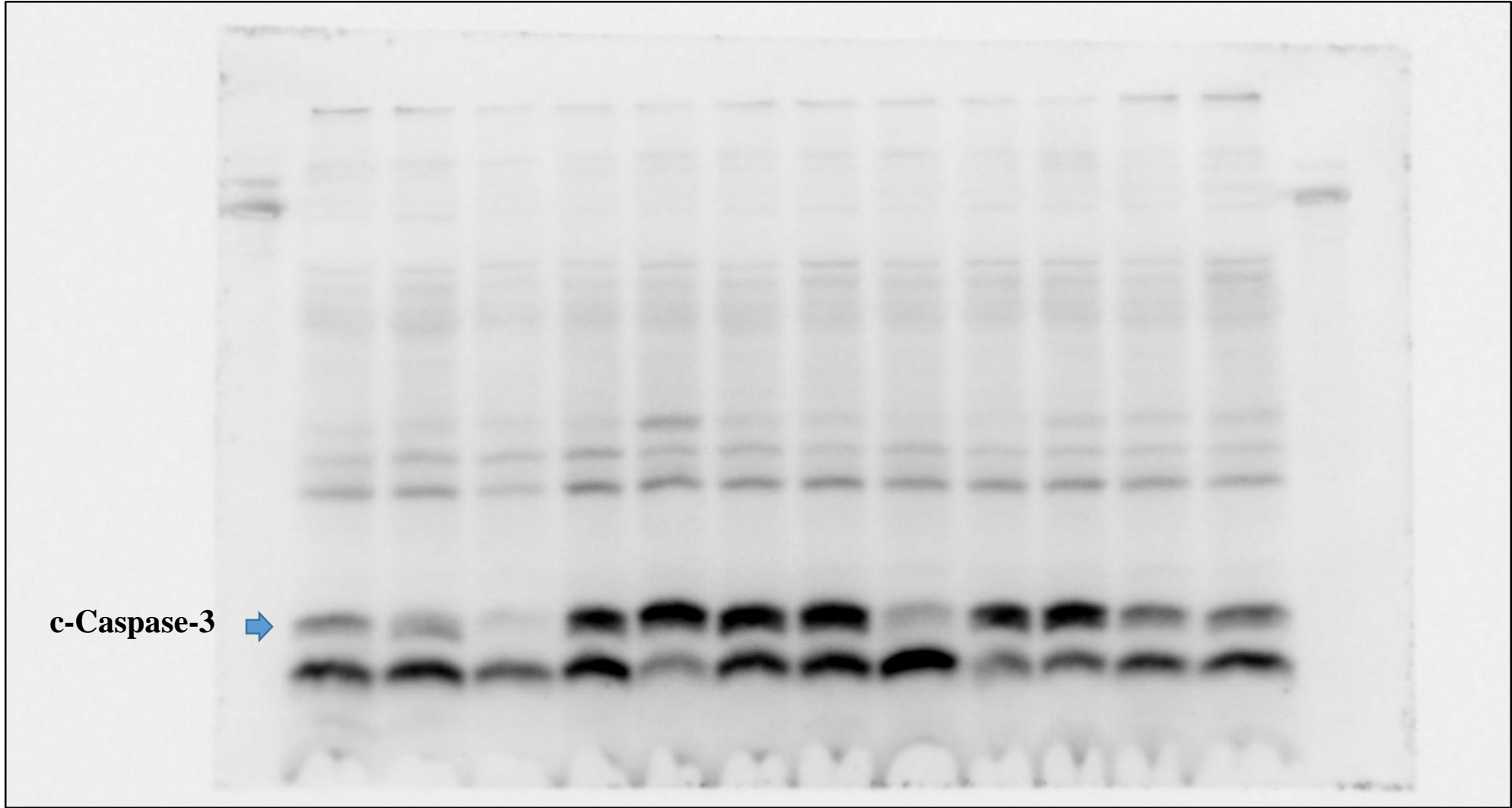

**T-Caspase-3** ➡

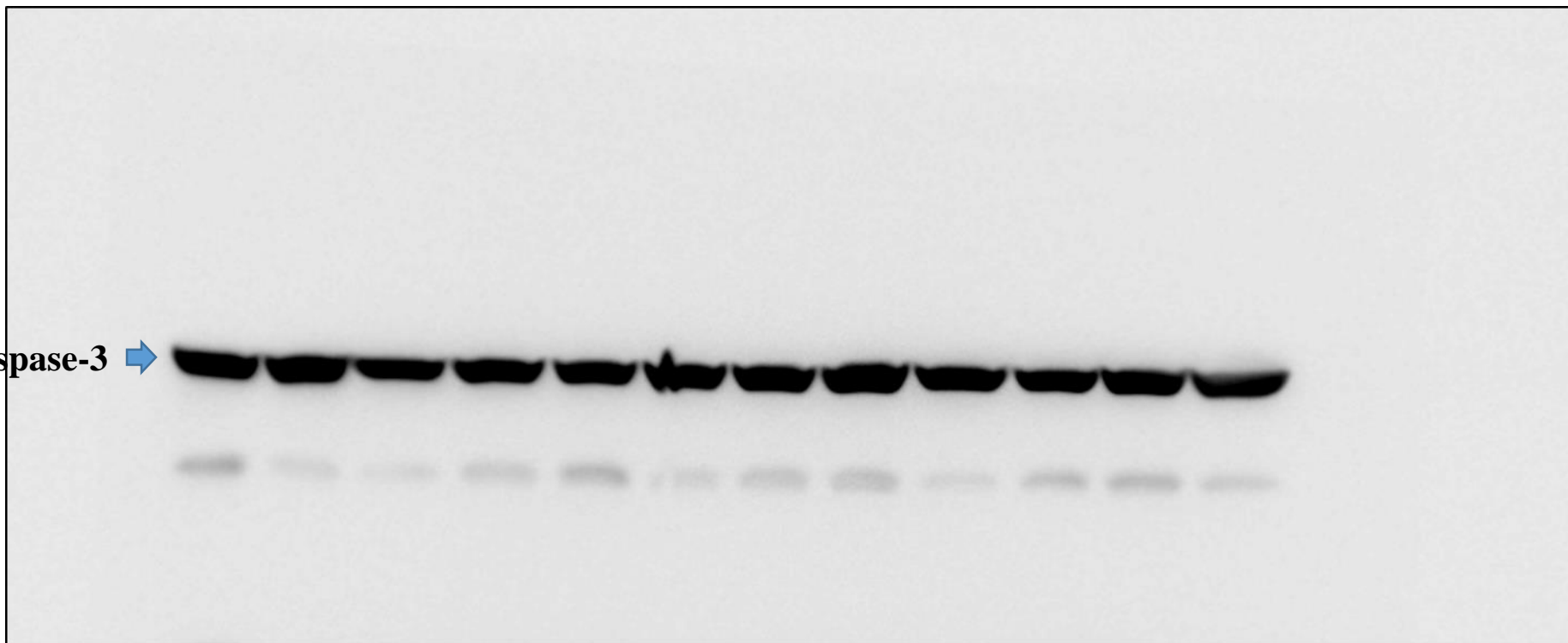

**Figure 4A**

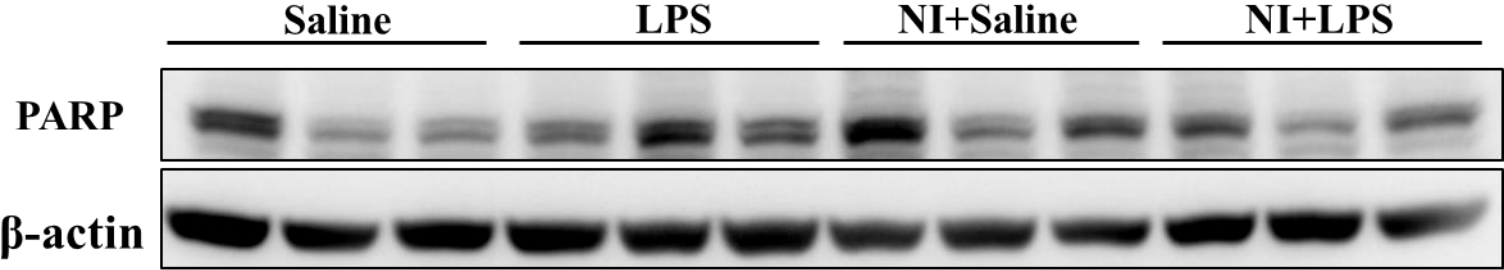

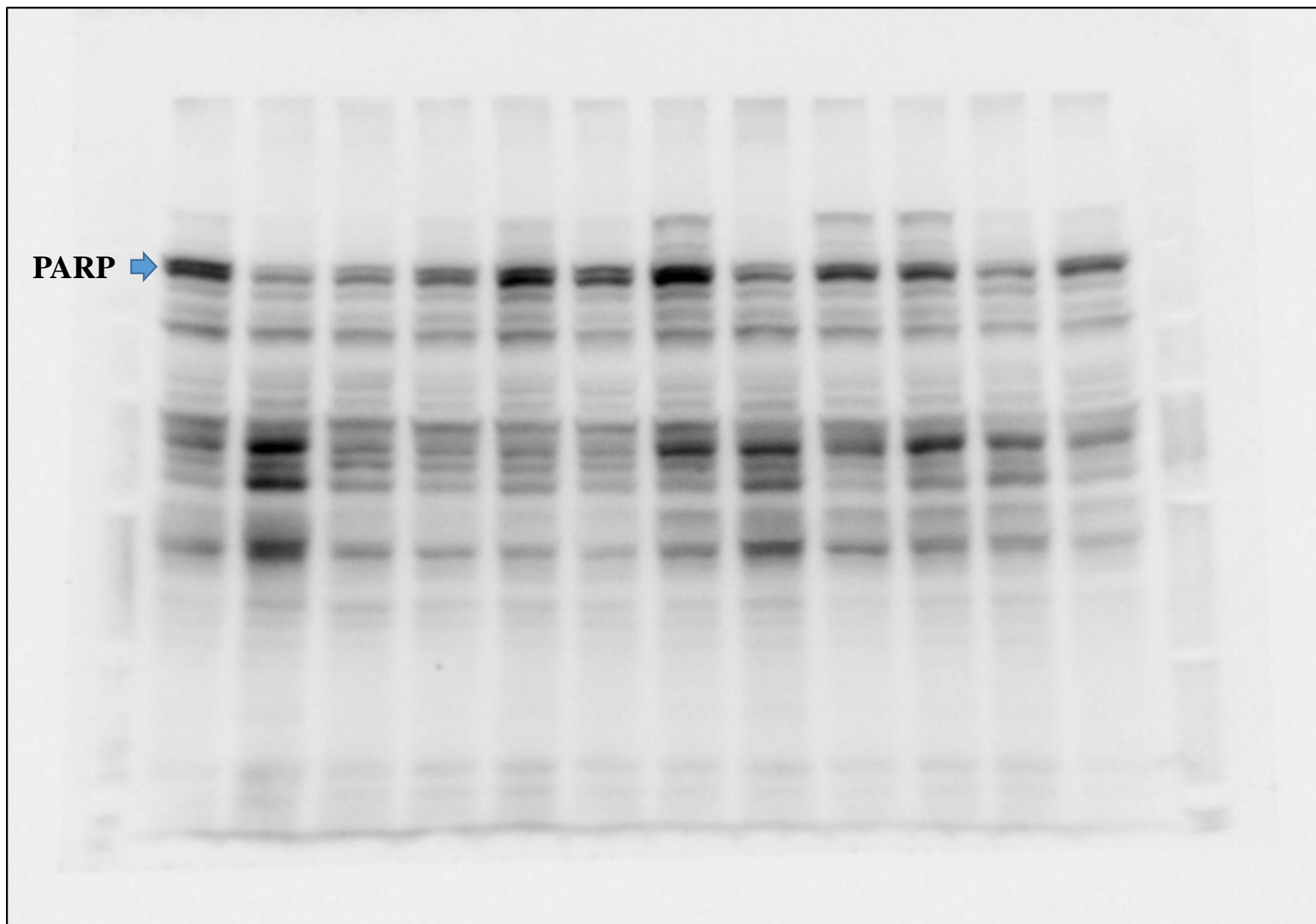

$\beta$ -actin →

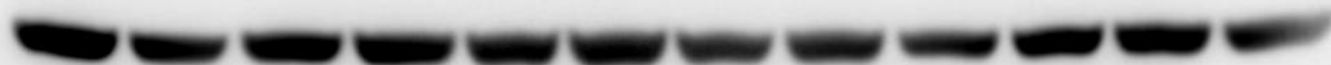

**Figure 5A**

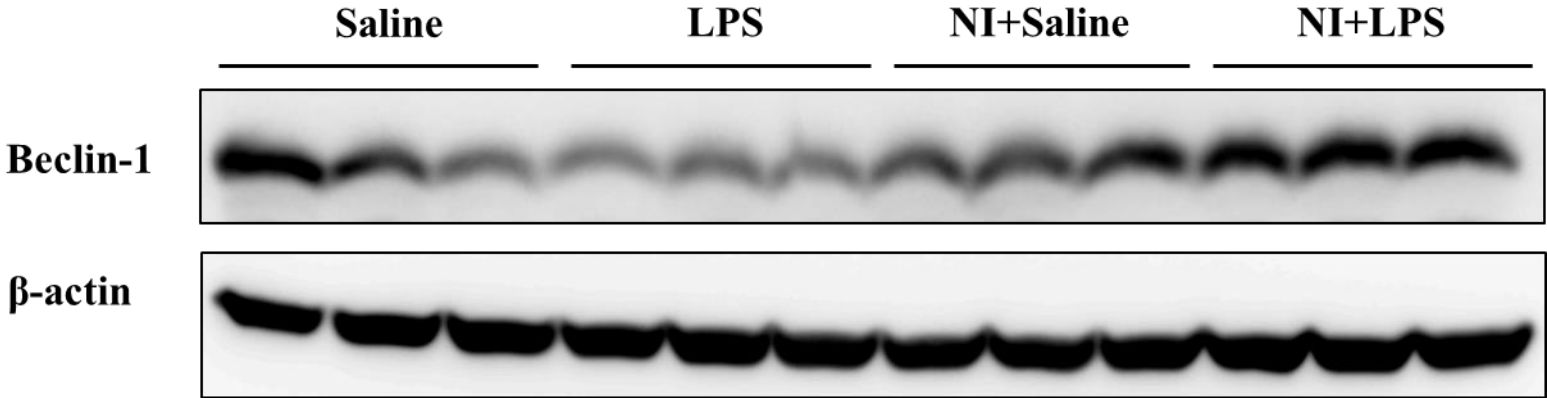

**Beclin-1** →

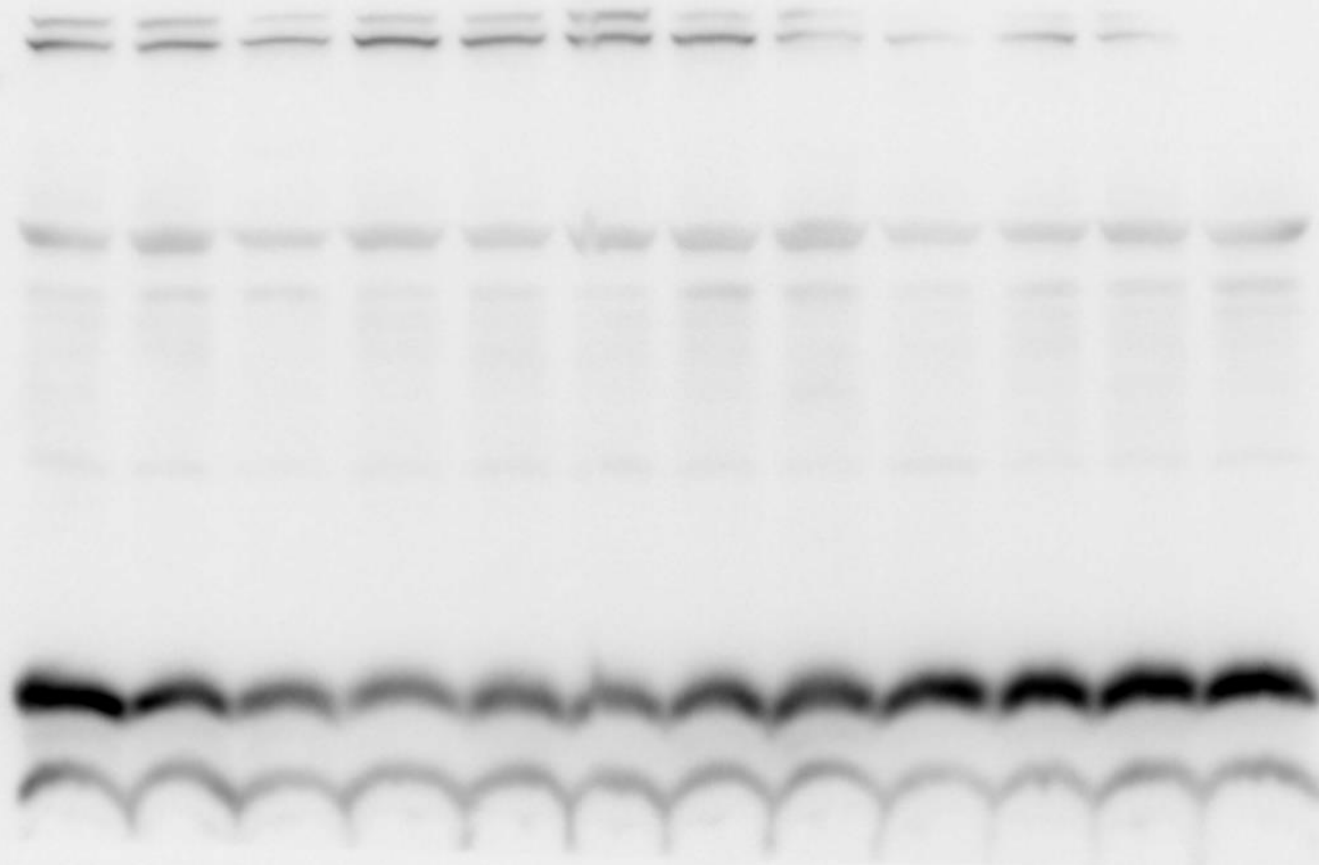

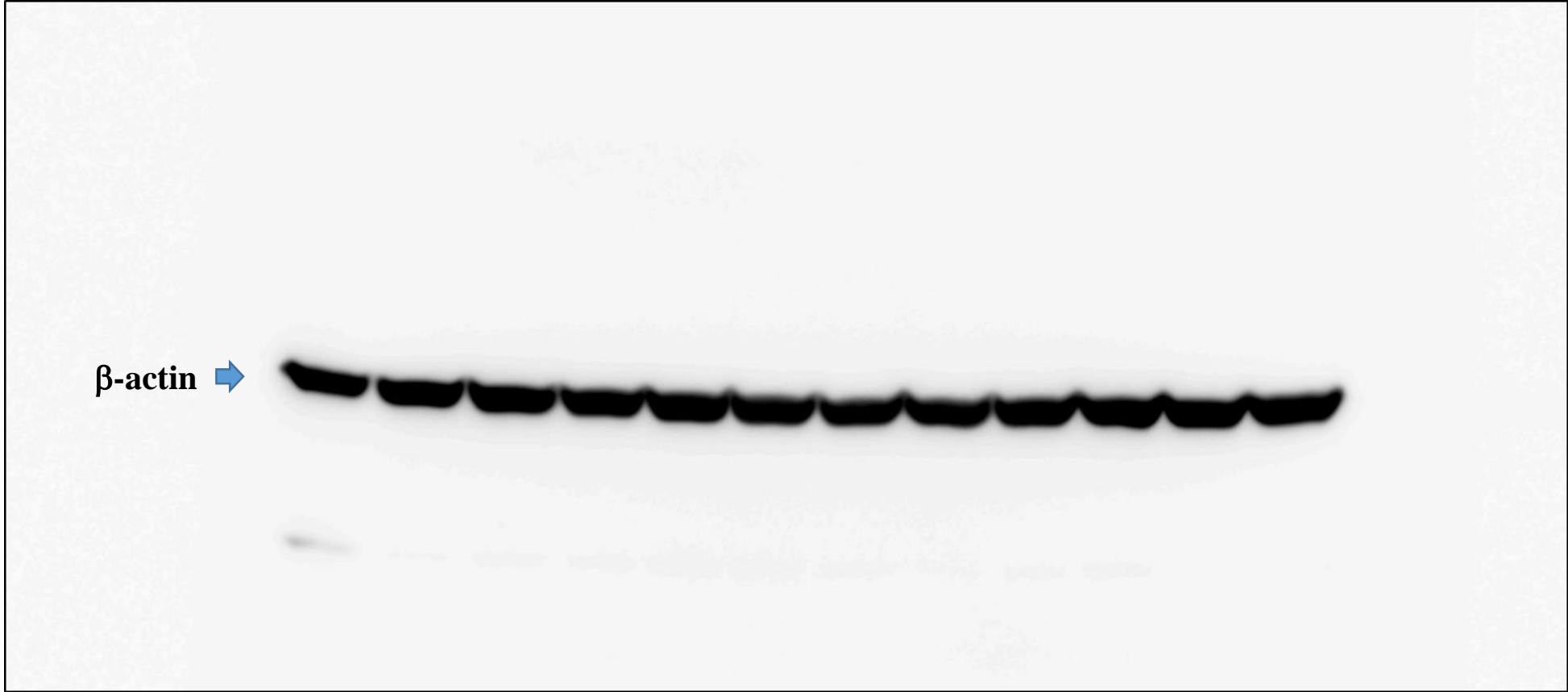

**Figure 6A**

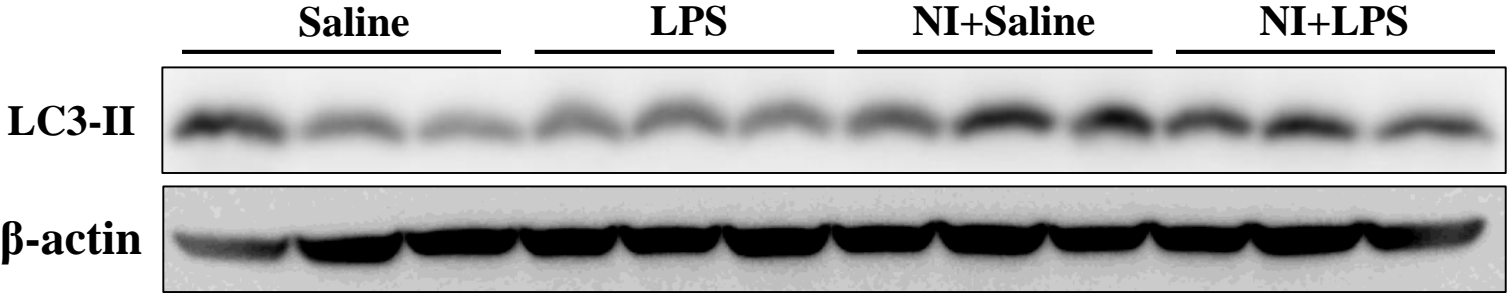

LC3-II →

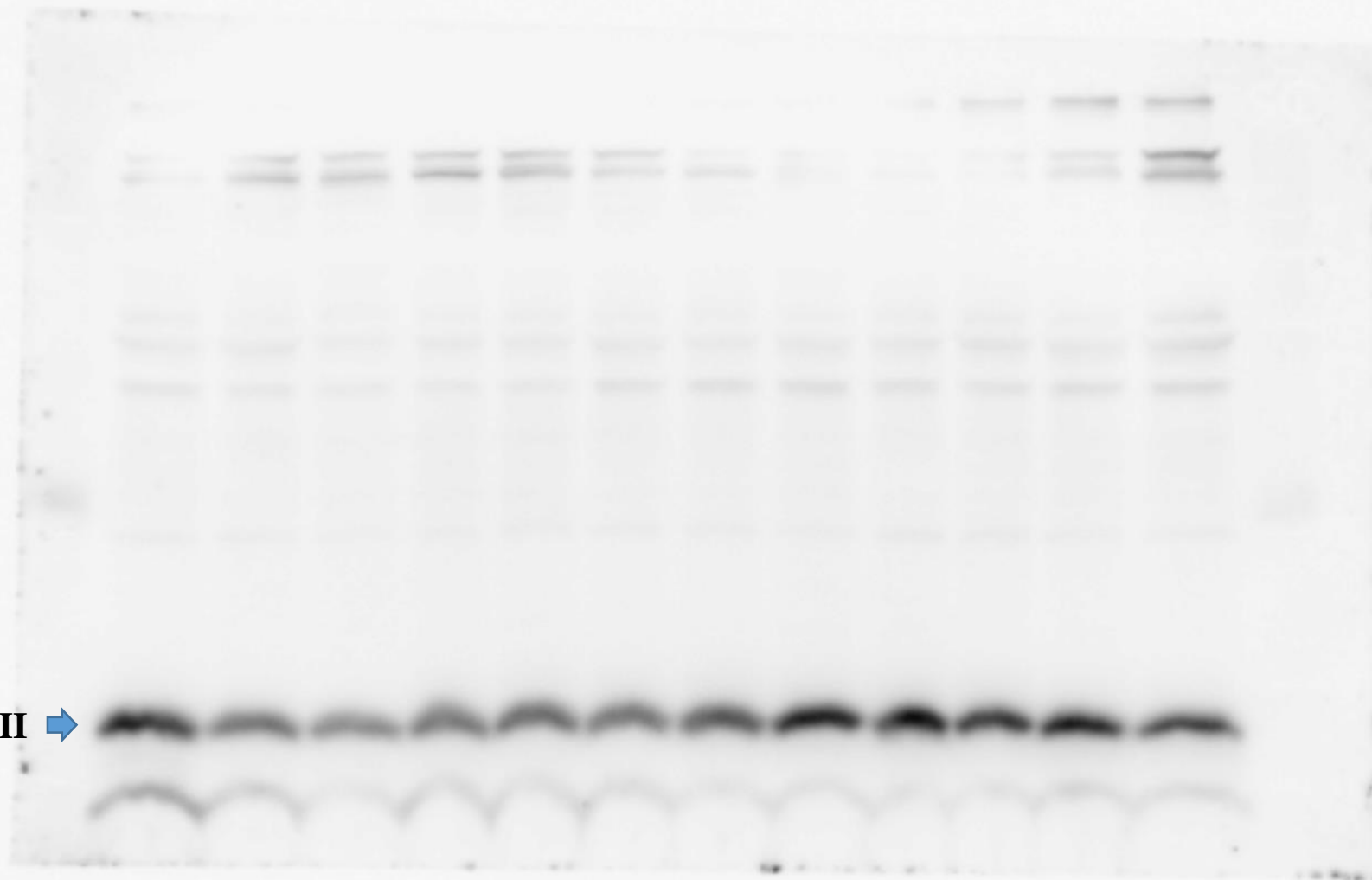

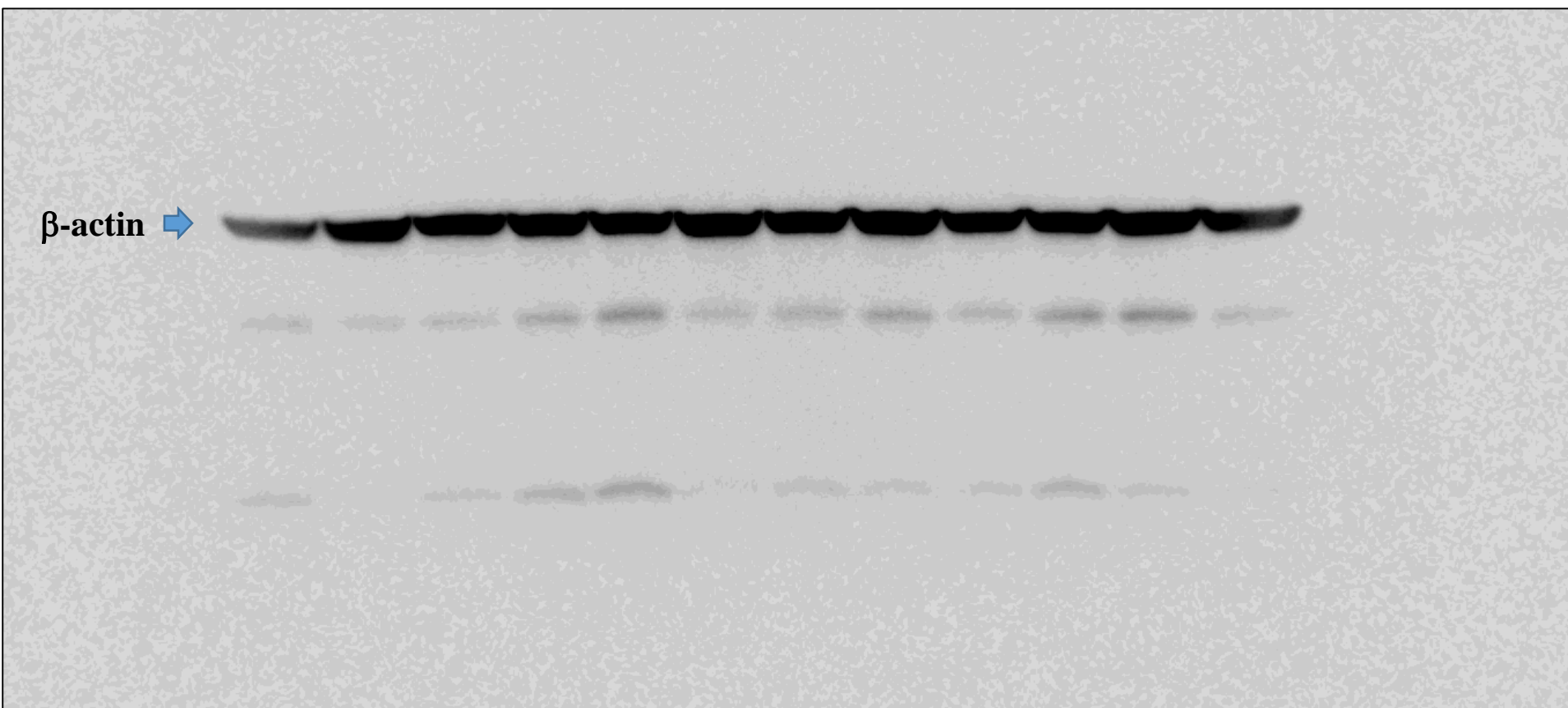

Supplement: S1 Raw images — (PDF) [file pone.0275748.s001.pdf]
